# Title, Table of Contents and Acknowledgements

**DOI:** 10.1080/26410397.2019.1705121

**Published:** 2019-12-27


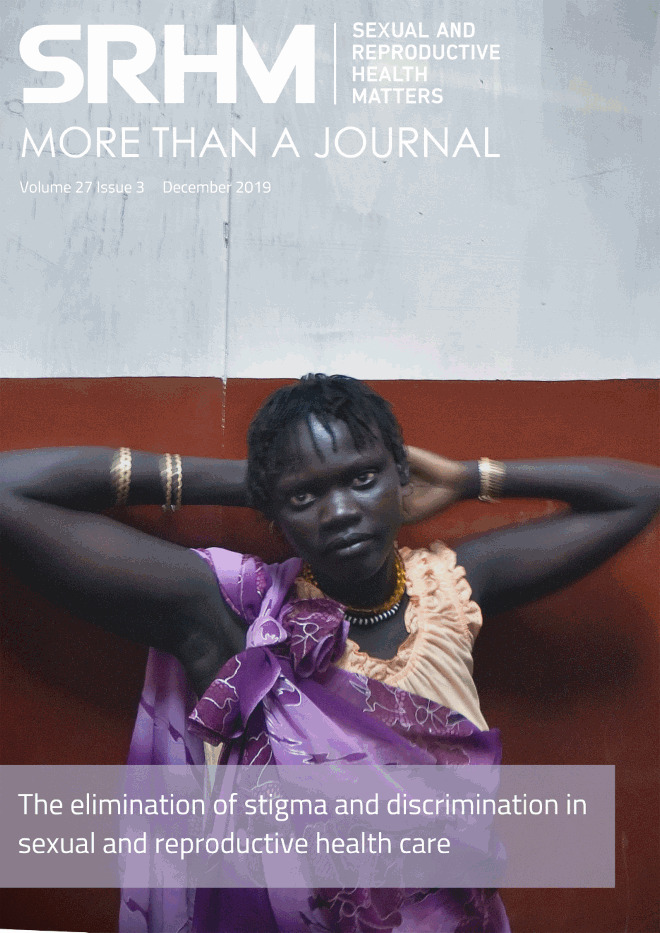


**Editorial**

1 *Julia Hussein, Laura Ferguson*

Eliminating stigma and discrimination in sexual and reproductive health care: a public health imperative

**Review article**

6 *Pauline Cullen, Elżbieta Korolczuk*

Challenging abortion stigma: framing abortion in Ireland and Poland

**Research articles**

20 *Ulrika Rehnström Loi, Beatrice Otieno, Monica Oguttu, Kristina Gemzell-Danielsson, Marie Klingberg-Allvin, Elisabeth Faxelid, Marlene Makenzius*

Abortion and contraceptive use stigma: a cross-sectional study of attitudes and beliefs in secondary school students in western Kenya

32 *Tricia Ong, David Mellor, Sabrina Chettri*

Multiplicity of stigma: the experiences, fears and knowledge of young trafficked women in Nepal

Correction to: Multiplicity of stigma: the experiences, fears and knowledge of young trafficked women in Nepal

50 *Shelly Makleff, Rebecca Wilkins, Hadassah Wachsmann, Deepesh Gupta, Muthoni Wachira, Wilson Bunde, Usha Radhakrishnan, Beniamino Cislaghi, Sarah E Baum*

Exploring stigma and social norms in women's abortion experiences and their expectations of care

65 *Sandra Salomé Fernández Vázquez, Josefina Brown*

From stigma to pride: health professionals and abortion policies in the Metropolitan Area of Buenos Aires

75 *Meghan Seewald, Lisa A Martin, Lina Echeverri, Jesse Njunguru, Jane A Hassinger, Lisa H Harris*

Stigma and abortion complications: stories from three continents

86 *Carmen H Logie, Moses Okumu, Simon P Mwima, Peter Kyambadde, Robert Hakiza, Irungu Peter Kibathi, Emmanuel Kironde, Joshua Musinguzi, Claire Uwase Kipenda*

Exploring associations between adolescent sexual and reproductive health stigma and HIV testing awareness and uptake among urban refugee and displaced youth in Kampala, Uganda

107 *Dulce Ferraz, Marcia Thereza Couto, Eliana Miura Zucchi, Gabriela Junqueira Calazans, Lorruan Alves dos Santos, Augusto Mathias, Alexandre Grangeiro*

AIDS- and sexuality-related stigmas underlying the use of post-exposure prophylaxis for HIV in Brazil: findings from a multicentric study

**Perspective**

122 *Clare Murphy, Verity Pooke*

Emergency contraception in the UK: stigma as a key ingredient of a fundamental women's healthcare product

**Bookshelf**

126 *Olivia Engle*

*From a Whisper to a Shout: Abortion Activism and Social Media.* By Elizabeth Arveda Kissling

**Editor-in-Chief:** Julia Hussein

**Chief Executive:** Eszter Kismödi

**Managing Editor:** Pete Chapman, Sarah Pugh

**Monitoring Editor:** Pathika Martin

**Communications Manager:** Jessica MacKinnon

**Communications Officer:** Alexane Bremshey

**Finance Manager:** Elisabeta Pashaj, Lance Stewart

**Operations Manager:** Edna Epelu

**Associate Editors:** Laura Ferguson, Nambusi Kyegombe, Emma Pitchforth, Mindy Jane Roseman, Nina Sun, Joyce Wamoyi

Peer reviewers:

Renu Addlakha, Sylvia Avon, Pierre Waldemar Brouard, Catherine Cansino, Roosbelinda Cardenas, Brenda Chizana, Kalysha Closson, Diane Cooper, Sarah Cowan, Laura Ferguson, Rebecca Fish, Dennis Francis, Jewel Gausman, Rakhi Ghoshal, Lesley Hoggart, Fauzia Huda, Alexandrina Iovita, Derina Johnson, Heidi Bart Johnston, Gary Jones, John Kingsley Krugu, Elmien Lesch, Carmen H Logie, Lucy Wangui Maina, Pam Lowe, Lucy Minayo, Suraya Mohamed, Simone Monteiro, Benita Moolman, Oliver Mweemba, Erica Marie Nelson, Theresa Ngoshe Nkole, Kathryn S Oths , Sara Parker, Susan Patterson, Erin Pearson, Mario Pecheny, Melisa Pamela Quispe-Ilanzo, Sarah Rominski, Michelle Sadler, Udo Schuklenk, Meghan Seewald, Arianne Shahvisi, Holly Donahue Singh, Edwin Van Teijlingen, Leela Visaria, Joyce Wamoyi, Jane Wilbur, Elspeth Wilson

Funding

SRHM’s work in 2019 has been supported by the Open Society Foundation and the Women's Refugee Commission.

Authors are responsible for the content of their articles which do not necessarily reflect positions or policies of the funders.

**Cover photo:** A young woman waiting in front of a consultation room in a hospital in Nasir, run by MSF (Médecins Sans Frontières). On the door it says “Free condoms are available here”. With the return of people from refugee camps in neighbouring countries, new health problems and diseases like HIV and AIDS have been introduced into local communities. International NGOs are trying to fight a looming health crisis in large parts of inaccessible South Sudan by raising awareness, distributing condoms and trying to change sexual behaviour.© Sven Torfinn / Panos

Translation:

Françoise de Luca-Lacoste translated abstracts from English to French and Lisette Silva translated abstracts from English to Spanish.

Copyright © 2019

**Sexual and Reproductive Health Matters**. This is an Open Access journal distributed under the terms of the Creative Commons Attribution License (http://creativecommons.org/licenses/ by/4.0/), which allows for sharing and adapting the work for any purpose, even commercially, provided appropriate credit is given with a link to the originally published item, a reference to the author(s) and links to their homepages, reference to the license under which the article is published and a link to this, as well as an indication of any changes that have been made to the original.

ISSN (Online) 2641-0397

SRHM in translation online

Selected papers from the SRHM journal are published in Arabic, Chinese, French, Hindi, Portuguese, Russian and Spanish. Go to: http://www.srhm.org/our-journals/

www.srhm.org / www.srhmjournal.org

Twitter @SRHMJournal

Facebook @SRHMJournal

